# Patterns of microbiome composition in tsetse fly *Glossina palpalis palpalis* during vector control using Tiny Targets in Campo, South Cameroon

**DOI:** 10.1128/spectrum.00935-24

**Published:** 2024-09-19

**Authors:** Calmes Ursain Bouaka Tsakeng, Tito Tresor Melachio Tanekou, François Sougal Ngambia Freitas, Inaki Tirados, Jean Marc Tsagmo Ngoune, Jude Daiga Bigoga, Flobert Njiokou, Charles Sinclair Wondji

**Affiliations:** 1 Centre for Research in Infectious Diseases (CRID), Yaoundé, Cameroon; 2 Department of Biochemistry, Faculty of Science, University of Yaoundé I, Yaoundé, Cameroon; 3 Department of Microbiology and Parasitology, Faculty of Science, University of Bamenda, Bamenda, Cameroon; 4 Department of Vector Biology, Liverpool School of Tropical Medicine (LSTM), Pembroke Place, Liverpool, United Kingdom; 5 Department of Parasites and Insect Vectors, Trypanosome Transmission Group, Trypanosome Cell Biology Unit, INSERM U1201, Institut Pasteur, Université Paris Cité, Paris, France; 6 Department of Animal Biology and Physiology, Faculty of Science, University of Yaoundé I, Yaoundé, Cameroon; USDA-ARS-NPRL, Dawson, Georgia, USA

**Keywords:** African trypanosomiases, vector control, tsetse fitness, microbiome

## Abstract

Novel vector control tools against African trypanosomiases require a deep understanding of the factors driving tsetse vector fitness or population resilience in their ecosystems. Following evidence of microbiota-mediated host fitness or traits shaping, including insecticide resistance in arthropod populations, we undertook a comparative study of the microbiota in wild-caught tsetse flies during vector control with deltamethrin-impregnated traps called Tiny Targets. The bacterial microbiome composition of tsetse flies collected before and after 6, 12, and 18 months of vector control were characterized using high-throughput sequencing of the V3-V4 hypervariable region of the bacterial 16S rRNA gene and compared. Overall, 48 bacterial genera and five phyla were identified. The primary symbiont *Wigglesworthia* dominated almost all the samples with an overall relative abundance of 71.76%. A significant increase was observed in microbiome diversities over the vector control with new taxa identified. Interestingly, few genera, like *Curvibacter* for instance, displayed a regularly increasing abundance, from 0.57% to 0.65%, 4.73%, and 8.57% after 6, 12, and 18 months of tsetse control, respectively. This study provided preliminary for further investigation into the role and mechanism of action of microbiota in tsetse fly fitness under selective pressure like insecticides.

**IMPORTANCE:**

The interest in vector control in the fight against African trypanosomiases has been reinforced in recent years, with the development of small insecticide-impregnated screens, known as “Tiny Targets”. As some tsetse biotopes are difficult to access for their installation, other tools are under consideration that involve using bacteria harbored by the tsetse vector to block the development of trypanosomes or impair the tsetse’s fitness in its natural environment. Several bacterial symbionts were previously described as important for tsetse fly development, and some like *Burkholderia* and *Citrobacter* also found in tsetse flies were found associated with insecticide tolerance in other arthropods. In this research, we found the bacterial genera, *Curvibacter* and *Acinetobacter,* increased in abundance in tsetse flies during vector control. These bacteria deserve further attention to determine if they can interfere with insecticides used to control tsetse fly populations.

## INTRODUCTION

African trypanosomiases caused by protozoan parasites of the genus *Trypanosoma* are transmitted to humans and other vertebrates by blood-sucking tsetse flies of the genus *Glossina*. The human disease known as human African trypanosomiasis (HAT or sleeping sickness) is a major public health problem in poor rural settings in sub-Saharan Africa, and the animal disease (AAT or nagana) is a severe constraint to the development of agriculture and livestock, with an estimated cost of US $4.5 billion per year (1). Human infection with *Trypanosoma brucei gambiense* causes a chronic form of sleeping sickness in west and central Africa, whereas *T. brucei rhodesiense* infection results in the acute form of sleeping sickness in east and southern Africa (2). During the last decades, efforts to control HAT through national control programs in relation to the World Health Organization(WHO) roadmap of 2020 disease elimination resulted in a decline in the incidence over the years from 10,000 cases reported in 2009 to the lowest records of 663 infected patients in 2020 (3). This decrease is the result of mass screening and treatment of populations, that have been limited by low coverage due to the difficult access to exposed populations in some settings but supported by vector control (4–6). Nevertheless, the lack of vaccines and the presence of animal reservoirs, which ensure the circulation of parasites at a relatively low level (7, 8) and failures in some tsetse control actions (9), have been the main obstacles to this fight.

Insect control has mainly relied on insecticide-based tools that are known to have a negative impact on the environment. New ecological-friendly tools under development involve using symbionts that are transmitted maternally to impair the development of parasites or other life traits (10). Previous studies have linked symbiotic bacteria to their host fitness or ability to survive with improved adaptability to the environment (11); other symbionts were shown to enhance immunity against pathogenic microorganisms in the host or increase host detoxification rates (12, 13). Also, interestingly, it was recently shown that particular microbiome compositions drive genomic adaptation in *Drosophila melanogaster* (14), suggesting that this interaction occurs in other insects. Several bacterial symbionts that the tsetse fly harbors were found to be important for its development, including *Wigglesworthia glossinidia*, the obligate tsetse fly mutualist, necessary for the fly’s fertility and immune response (15); the secondary symbiont *Sodalis glossinidius*, found to be involved in trypanosome establishment (16, 17); and *Wolbachia* sp, which acts on the reproductive process of tsetse flies by inducing cytoplasmic incompatibility (18–20). Recently, a metagenomic approach helped identify more bacterial taxa in these flies (21–23). Although the potential functions of these bacteria are not yet elucidated, some taxa like *Serratia*, *Ralstonia,* or *Staphylococcus* were statistically associated with mature infection to trypanosomes, that is, trypanosomes found in tsetse mouthparts and ready to be transmitted to the next host. Nevertheless, other bacteria taxa also identified in tsetse flies were previously shown to be associated with important biological functions in other organisms. For example, studies on agricultural pests showed that the presence of *Burkholderia* in *Riptortus pedestris* (24) and *Citrobacter* in *Plutella xylostella* gut (25) increased insecticide tolerance in the host. Moreover, the microbial composition of *Anopheles albimanus* differed between fenitrothion-susceptible and -resistant strains, and mosquitoes exposed to permethrin and cypermethrin were shown to harbor different microbial compositions relative to non-exposed mosquitoes (26, 27). These studies suggest the potential mediating role of gut bacteria in insect fitness, and we hypothesize that the diversity and composition of tsetse fly microbiome may vary during their control with insecticides.

In this study, we compared bacteria communities of the tsetse fly *Glossina palpalis palpalis* before and during the vector control using insecticide-impregnated Tiny Targets to understand how the composition and structure of tsetse microbiota could influence vector control and identify bacteria that may drive tsetse fitness.

## MATERIALS AND METHODS

### Study area

The study was conducted in Campo (2°20′N, 9°52′E) trypanosomiases focus in the South Region of Cameroon. Campo is located on the Atlantic coast, sharing with Equatorial Guinea, a natural border that is the river Ntem. The climate is of equatorial type with two rainy seasons and two dry seasons yearly, and there is a dense hydrographic network with several rivers, swampy areas, and marshes. The main activities of Campo inhabitants are fishing, hunting, and farming, which exposes them to tsetse bites and HAT transmission. In this region, the composition of wild fauna is highly diversified (28). Previous studies reported the presence of several tsetse fly species, namely *Glossina palpalis palpalis*, *Glossina pallicera*, *Glossina caliginea*, and *Glossina nigrofusca* (29, 30). A small-scale tsetse control intervention was initiated in 2020 using insecticide-impregnated Tiny Targets (31) in the frame of the PIIVeC project (Partnership for increasing the impact of Vector Control - https://essentials.lstmed.ac.uk/piivec-0).

### Tsetse collection surveys

Tsetse flies were sampled using pyramidal traps (32) in July 2019 for pre-intervention surveys before starting vector control in January 2020 and every 6 months during vector control with Tiny Targets, that is, August 2020, January 2021, and August 2021 (map and more description on sampling in Melachio Tanekou et al. (33)). Traps were set in various tsetse fly favorable biotopes (mostly water points and riverbanks), and the geographical coordinates of each were recorded with a global positioning system. Flies were collected once a day for three consecutive days between 12:00 p.m. and 02:00 p.m.. The species, sex, and teneral status of each collected tsetse fly were identified morphologically (34). Tsetse flies were then sterilized twice with 0.5 N sodium hypochlorite and rinsed twice with distilled water to eliminate potential bacterial contaminants from the environment. The flies were then conserved in labeled microtubes containing ethanol 95%. Once in the laboratory, these samples were stored at −20°C until DNA extraction and further analyses.

### DNA extraction

DNA was extracted from whole fly bodies using the LIVAK protocol (35) modified as described in Bouaka Tsakeng et al. (23). Briefly, the tubes containing tsetse flies were left opened at room temperature to evaporate the alcohol, and 500 µL of LIVAK solution were added into each tube (LIVAK: 1.6 mL NaCl 5M; 5.48 g Sucrose; 1.57 g Tris; 10.16 mL EDTA 0.5M; 2.5 mL 20% SDS; distilled water to 100 mL total volume). The contents of each tube were crushed using adapted tube pestles, and the tubes were incubated at 65°C for 30 minutes. Then, 70 µL of 8 M potassium acetate solution were added, and tubes were homogenized, incubated on ice for 30 minutes, and centrifuged at 13,500 rpm for 20 minutes. The aqueous upper phase with nucleic acids was transferred into newly labeled Eppendorf tubes, and 1 mL of absolute ethanol was added to precipitate the nucleic acids. After homogenization, tubes were centrifuged at 13,500 rpm for 15 minutes. The pellet obtained was washed twice with 200 µL of 70% ethanol. The alcohol was completely removed after centrifugation, and tubes were left open about 1 hour to evaporate residual alcohol. The pellets were finally resuspended in 100 µL distilled water and stored at −20°C for subsequent molecular analyses.

### Determination of flies’ microbiome composition

#### Library preparation and sequencing

Sequencing was performed with DNA from 148 individual flies using the Illumina MiSeq platform (*Polo d’ Innovazione di Genomica Genetica e Biologia,* https://www.pologgb.com/). Of these tsetse flies, 13 were captured before vector control and 45, 45, and 45 after 6, 12, and 18 months of vector control with Tiny Targets, respectively. The V3–V4 region of the bacterial 16S rRNA gene was sequenced using two degenerated primers, with the respective forward and reverse primers with Illumina overhang adapters: 5′-TCGTCGGCAGCGTCAGATGTGTATAAGAGACAGCCTACGGGNGGCWGCAG-3′ and 5′-GTCTCGTGGGCTCGGAGATGTGTATAAGAGACAGGACTACHVGGGTATCTAATCC-3′ (36, 37). The first sequencing step was amplicon generation with PCR using a 2× KAPA HiFi HotStart Ready mix (KAPA Biosystems), with products of ~550 bp that were verified using a Bioanalyzer. Then, PCR products were purified using AMPure XP (Beckman Coulter Genomics) beads to remove free primers and primer dimers, and 5 µL of purified products was used to attach dual multiplexing indices (i5 and i7) and sequencing adapters as recommended by the manufacturer. The ~630 bp (2 × 300 bp) sequences obtained were normalized and used to construct the pooled libraries, which were denatured and loaded on the Illumina MiSeq flow cell (23, 37)

### Processing of the sequencing data

Illumina MiSeq reads were analyzed using Mothur v.1.44.3, following a pipeline described by Kozich et al. (38) and modified by Bouaka Tsakeng et al. (23). Briefly, forward and reverse demultiplexed paired-end reads were merged to contiguous sequences for each individual fly, and primers were trimmed, followed by quality filtering that removed all merged reads containing ambiguous bases. The data set was automatically screened to identify all unique sequences, and the number of sequences of each type was counted and used to generate a file summarizing those numbers for all the flies. Unique sequences were aligned against the SILVA v.123 reference database for their identification, and the data set was filtered to eliminate unique sequences with an abundance lower than 0.01% probably issued from sequencing errors. Also, highly similar sequences (up to one difference at each 100 base pairs) were pre-clustered, and chimeric sequences, or those classified as eukaryotes or mitochondria (probably from fly DNA), chloroplasts, or unknown, were removed. A distance matrix was built between the remaining sequences, and these later were clustered and classified into operational taxonomic units (OTUs). These OTUs were used to generate an OTU table that consisted of individual flies with all the OTUs they harbor, as well as their abundances.

### Statistical analyses

Ahead of comparative analyses, rarefaction curves were drawn after normalizing the reads to ensure the sequencing depth was enough to describe almost (if not all) taxa present in all individual flies. Four groups of tsetse flies of the sub-species *Glossina palpalis palpalis* were considered: flies captured before the vector control started and flies captured after 6, 12, and 18 months of vector control with deltamethrin-impregnated Tiny Targets. Alpha diversity was estimated with the Shannon diversity index (H) and compared between sampling periods using Wilcoxon signed-rank test. Bacterial microbiome composition was compared across sampling periods over the vector control with principal components analysis (PCA) using the Bray-Curtis dissimilarity analyses and ordination plots, and the differences were tested using permutational multivariate analysis of variance (PERMANOVA). Core microbiomes were computed and compared among fly groups using a Venn Diagram. Finally, differential abundance testing was performed to search potential taxonomic groups that caused the differences observed between tsetse fly groups and can serve as biomarkers associated with tsetse fly fitness during vector control with insecticide-impregnated Tiny Targets. All analyses and plots were done in the R environment (39), using a set of packages that worked in synergy, that is, “phyloseq” (40), “ggplot2” (41), “microbiome” (42), “vegan” (43), “knitr” (44), “ape” (45), “ggpubr” (46) “dendextend” (47), "VennDiagram" (48), and “DESeq2” (49).

## RESULTS

### Sample characteristics and 16S rRNA sequencing reads

Illumina sequencing of the V3–V4 hypervariable region of the bacterial 16S rRNA amplicons yielded a total of 15,607,674 raw sequence reads from 148 individual field-collected *Glossina palpalis palpalis* flies (13 before Tiny Targets implementation and 45 after 6, 12 and 18 months of vector control, respectively). After removing chimeric and other non-bacterial sequences, and quality filtering to remove bacterial OTUs with less than 0.01% abundance, a total of 7,659,379 sequences were obtained. From the analysis of the four groups and removal of outliers and contaminants, the sample rarefaction curves showed that the sequencing depth was enough for subsequent analysis (Fig. S1), with a plateau observed at around 4,000 reads while all the samples had more than 40,000 reads each.

### General composition of tsetse microbiome according to sampling periods

A total of 111 bacterial OTUs were detected and belonged to five phyla and 48 genera (Table S1). Most of the sequences were identified as belonging to the phylum *Proteobacteria* (96.69%) and were present in all 148 samples. The relative abundance of other bacteria phyla described was 2.48% for *Firmicutes*, 0.16% for *Chlamydiae*, 0.05% for *Acidobacteria*, 0.01% for *Bacteroidetes,* and 0.60% of sequences that could not be classified in a particular phylum.

**Fig 1:**
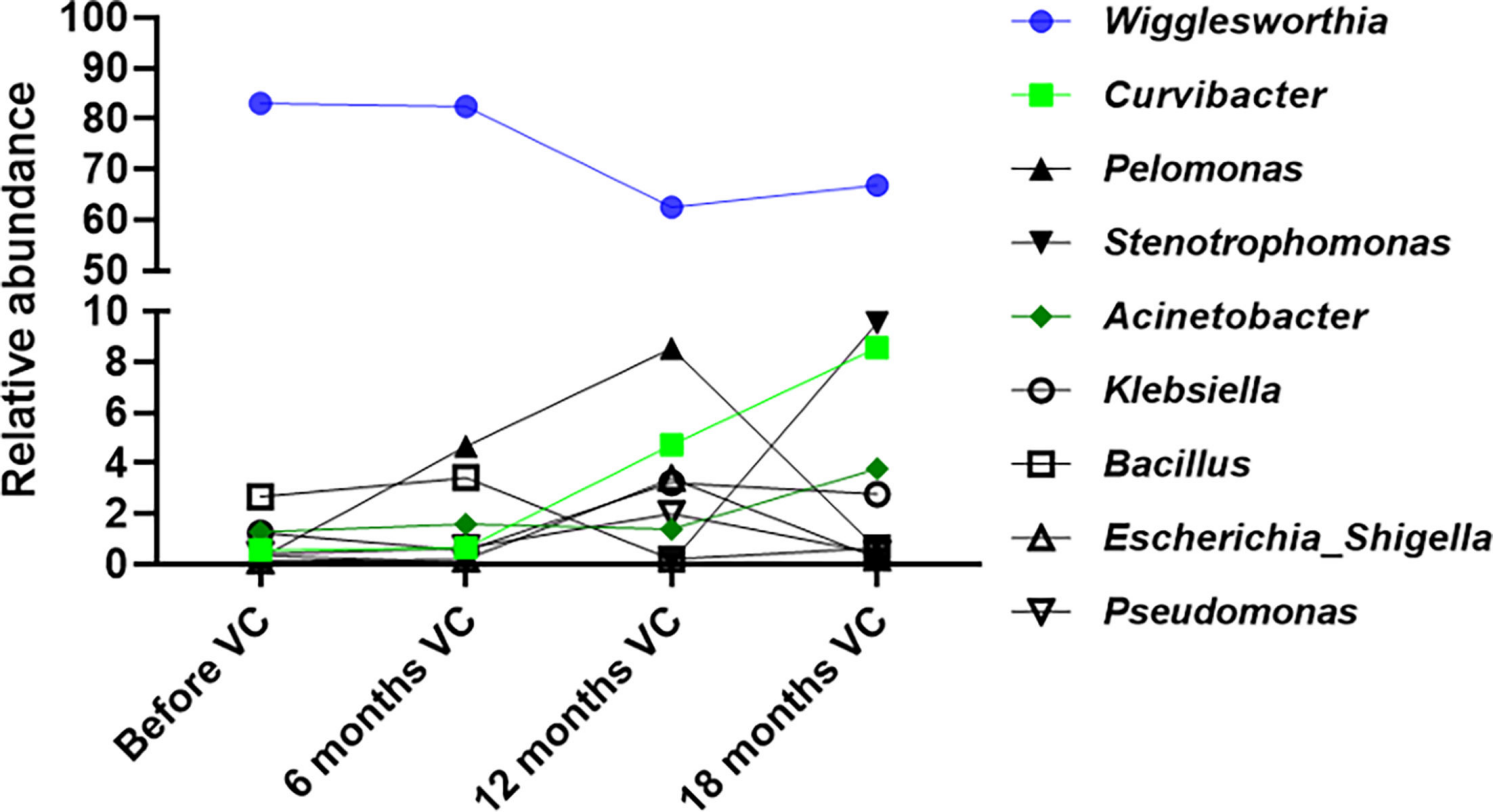
Variation of relative abundance of the nine most abundant bacteria genera in tsetse flies captured during the vector control (VC).

At the genus level, the most abundant bacterial genus in almost all flies was *Wigglesworthia,* the primary symbiont of tsetse flies (Table S2). Its relative abundance of 83.07% observed before the vector control decreased to 82.45%, 62.57%, and 66.87% after 6, 12, and 18 months of vector control, respectively. The other abundant genera found were *Curvibacter*, *Pelomonas*, *Stenotrophomonas*, *Acinetobacter*, *Klebsiella*, *Bacillus*, *Escherichia_Shigella* (which were highly similar in their V3-V4 sequences and could not be distinguished), and *Pseudomonas*. These bacteria showed a general increasing trend over the vector control period, particularly *Curvibacter*, that went from 0.57% before vector control, to 0.65, 4.73, and 8.57 after 6, 12, and 18 months of vector control, respectively (Fig. 1).

### Microbial community dynamics between sampling periods

**Fig 2:**
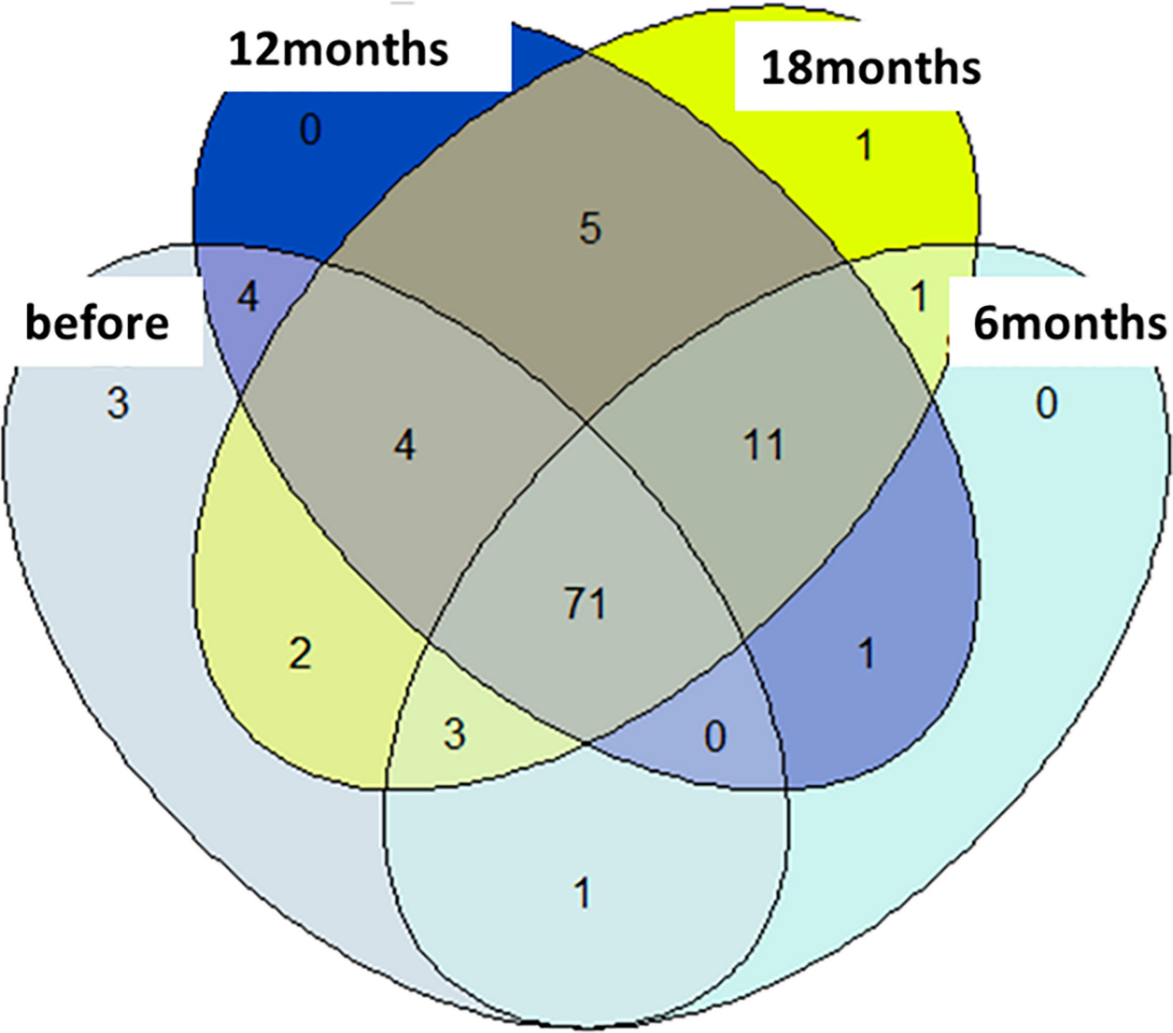
Venn diagram comparing the number of OTUs present in different sampling periods.

Of the 111 OTUs identified, 88 were found in flies collected before the installation of the screens, 88 were also found in flies collected 6 months later, whereas 96 and 98 were found in flies collected 12 and 18 months later, respectively (Fig. 2). Five OTUs were unique to flies collected before vector control, one was unique to flies collected 18 months after implementation of control, and 71 were common in all sample groups.

Of the five bacterial phyla identified, the relative abundance of *Firmicutes* showed a significant reduction during the vector control from 4.53% before vector control to 3.97%, 1.23%, and 1.63%, respectively, after 6, 12, and 18 months of vector control (*P*-value = 0.02). This decrease in *Firmicutes* abundance was concomitant to an increase in the relative abundance of *Proteobacteria* from 92.89% before to 95.78%, 97.34%, and 98.08%, respectively (*P*-value < 0.0001). There was no significant difference in the relative abundance of *Bacteroidetes* and *Chlamydiae* during the vector control (*P*-value = 0.08 and 0.13, respectively).

**Fig 3:**
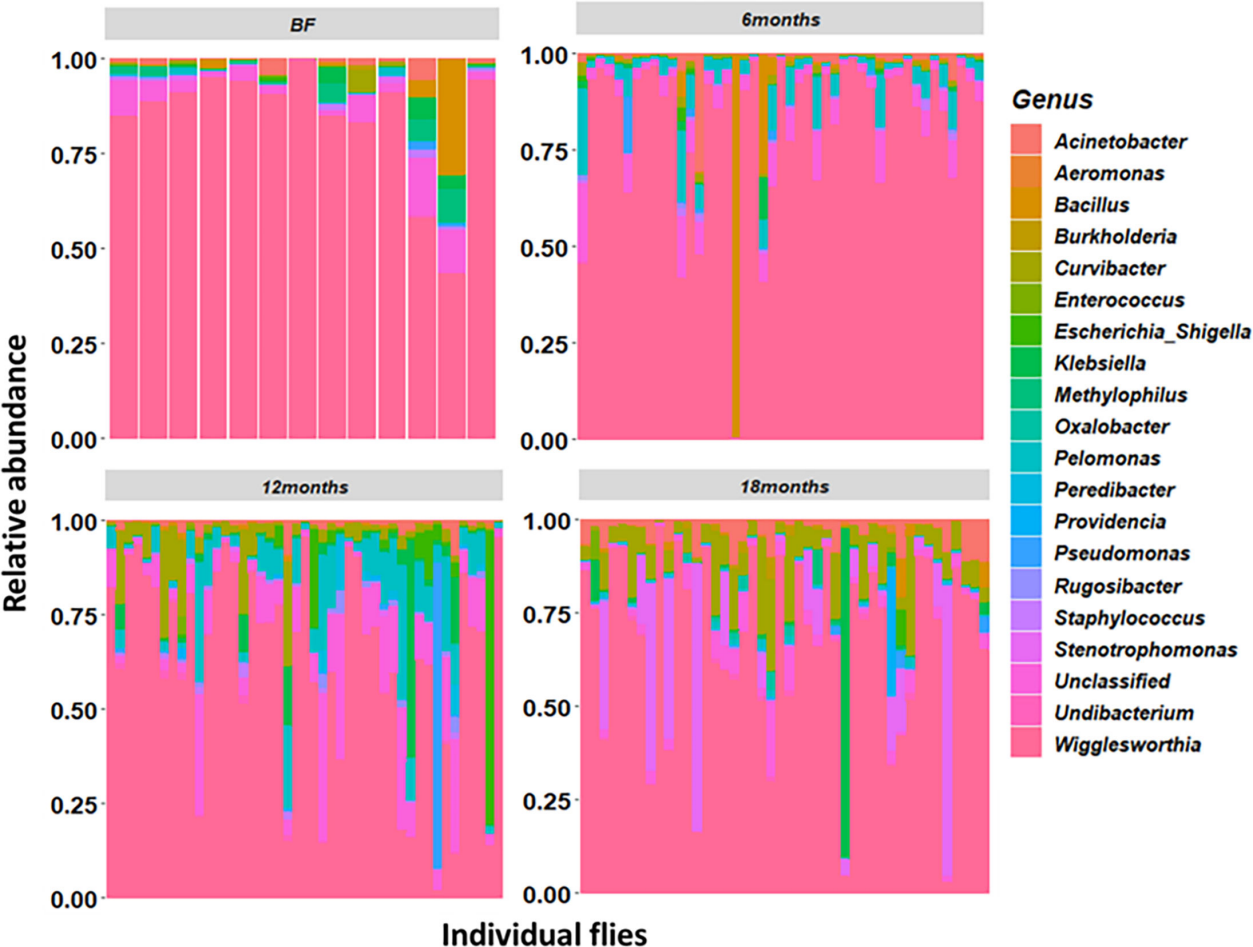
Map showing the relative abundance of the 20 most abundant bacterial genera in tsetse flies captured before the vector control (BF) and after 6, 12, and 18 months of tsetse control with Tiny Targets.

Some bacteria genera showed differential compositions during the vector control (Fig. 3; Table S1). For example, the most significant changes were observed in the primary symbiont *Wigglesworthia,* which displayed abundances of 83.07% and 82.45% before and after 6 months of vector control, respectively, but which significantly dropped to 62.57% and 66.87% (*P*-value < 0.001) at the 12th and 18th months, respectively. *Curvibacter* showed a significant increase (*P*-value = 0.001) in abundance from 0.57% to 0.65%, 4.73%, and 8.57% after 6, 12, and 18 months of tsetse control, respectively. The abundance of *Pelommonas* also increased from 0.27% to 4.67% (*P*-value < 0.001) and 8.55% (*P*-value < 0.001) after 6 and 12 months but dropped to 0.61% (*P*-value < 0.001) at the 18th months. Overall, the relative abundances of 11 of the 48 bacteria genera identified were significantly different between sampling periods (*P*-value < 0.05). Some bacteria taxa such as *Cupriavidus* and *Veillonella* were only found in tsetse flies collected before the Tiny Targets implementation. *Lactobacillus* was found only in flies collected after 6 months of vector control, whereas *Chromohalobacter* was found only in flies collected after 12 months and *Alishewanella* and *Oxalobacter* after 18 months.

### Microbiome diversity in tsetse flies over the vector control

**Fig 4:**
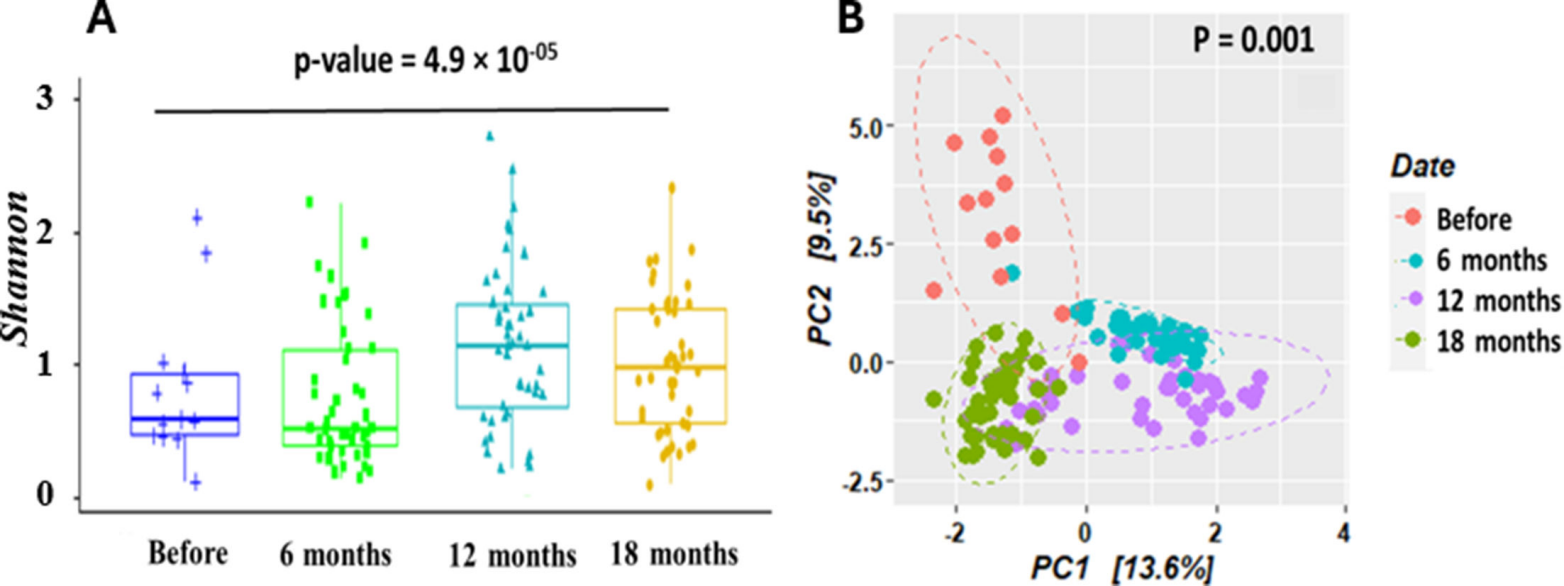
Bacterial diversity in tsetse fly (**A**) and distribution of tsetse fly samples according to their bacterial composition based on principal coordinates analysis using Bray-Curtis index (**B**) according to sampling periods.

The microbiome diversity in flies varied throughout the vector control with significant differences between some sampling periods (Fig. 4A). Indeed, although the alpha diversity estimated with the Shannon index did not vary after the first 6 months of control (H = 0.45 before and H = 0.44 after 6 months, *P*-value = 0.35), the diversity significantly increased to H = 1.24 twelve months later (*P*-value < 0.001), followed by a slight decrease to 0.99 after 18 months.

Regarding beta diversity, the structure of the flies’ microbiome composition showed a great heterogeneity between sampling periods as shown by the clear clustering of flies obtained by PCA performed using the Bray-Curtis dissimilarity index (Fig. 4B). This dissimilarity observed during the vector control implementation is supported by the permutational analysis of variance (PERMANOVA), showing a significant difference in the composition of the fly microbiota between the different sampling periods (R^2^ = 0.16; *P*-value = 0.001).

**Fig 5:**
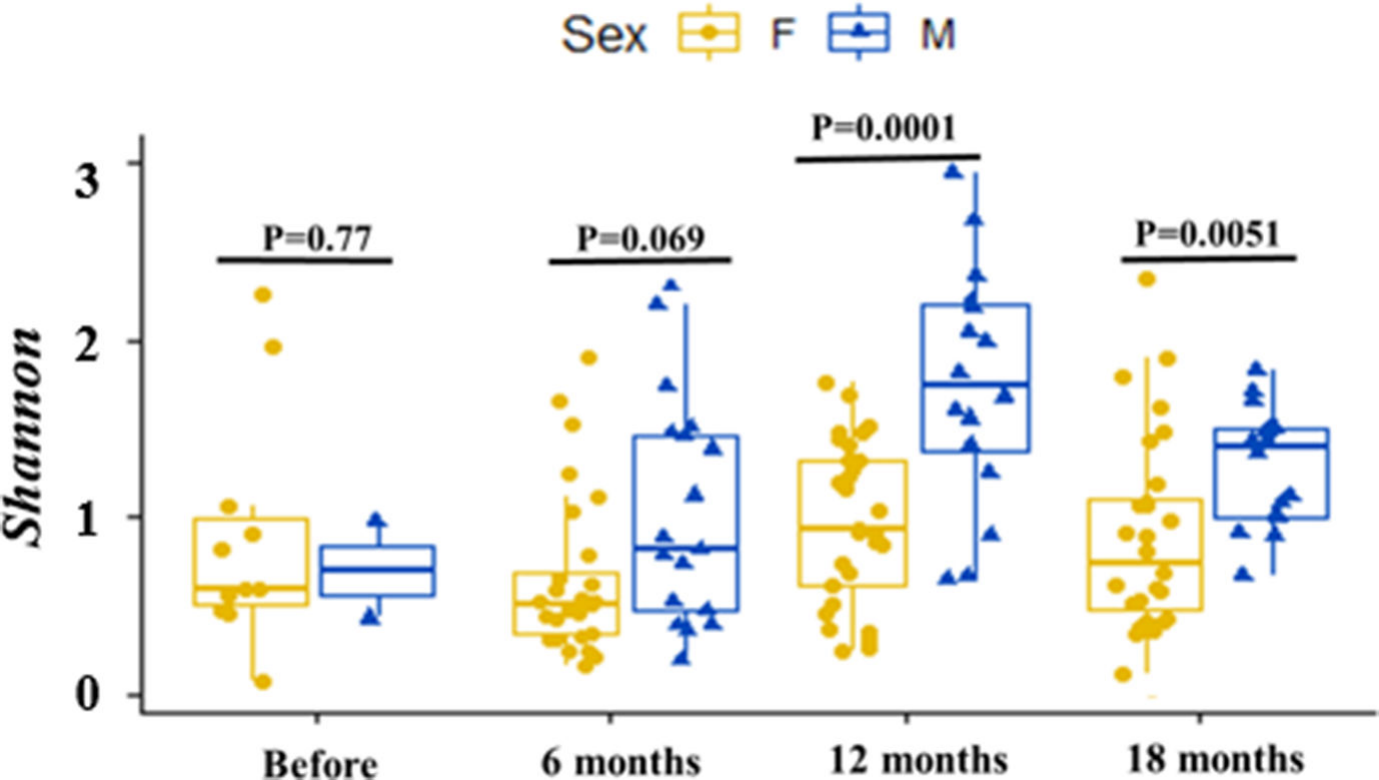
Bacterial diversity in male and female tsetse flies during vector control (F: female; M: male).

In addition, the diversity of the gut microbiota differs between male and female flies after 12 and 18 months of vector control (*P*-value = 0.0001 and 0.0051, respectively), where the α-diversity appears to be greater in males compared with females whatever the sampling periods (Fig. 5).

**Fig 6:**
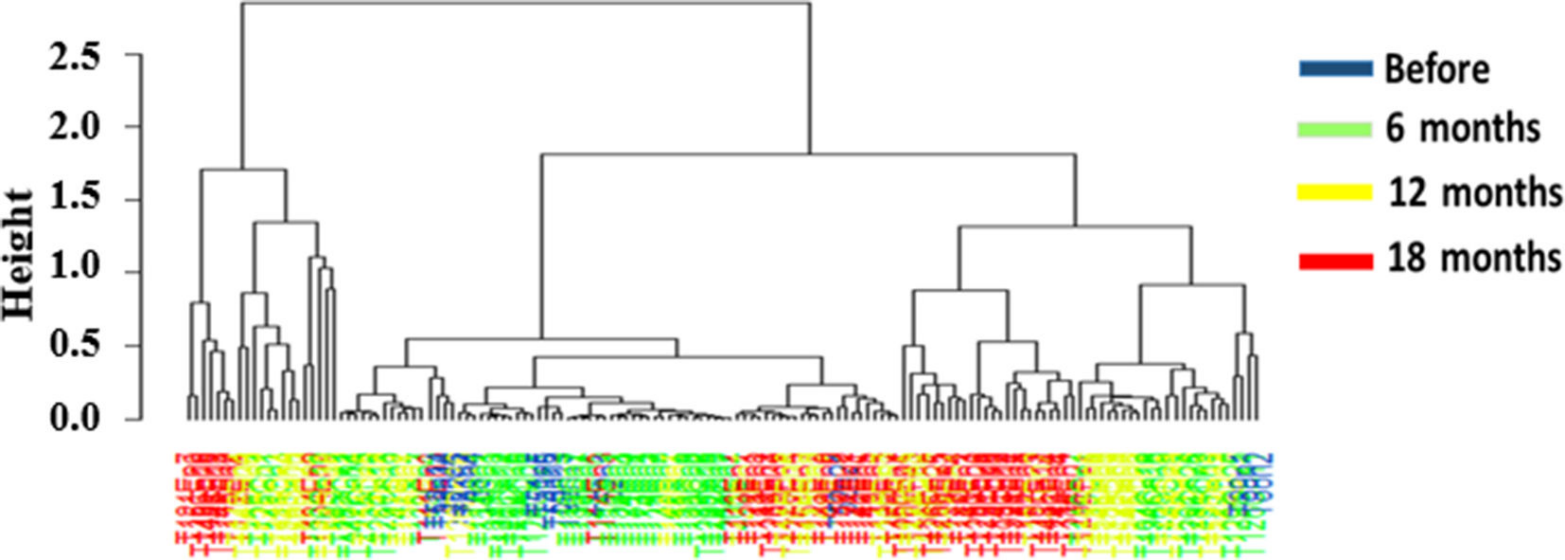
Hierarchical cluster dendrogram based on Bray-Curtis Index values, showing the relationship between different tsetse bacterial communities and sampling periods.

Furthermore, the dendogram of hierarchical clustering using the Bray–Curtis dissimilarity index showed that samples captured after 18 months of target implementation formed two main clusters slightly separated from samples from other periods. However, no clear high level of clustering was observed (Fig. 6).

### Differential abundance of bacteria taxa over the vector control

**Fig 7:**
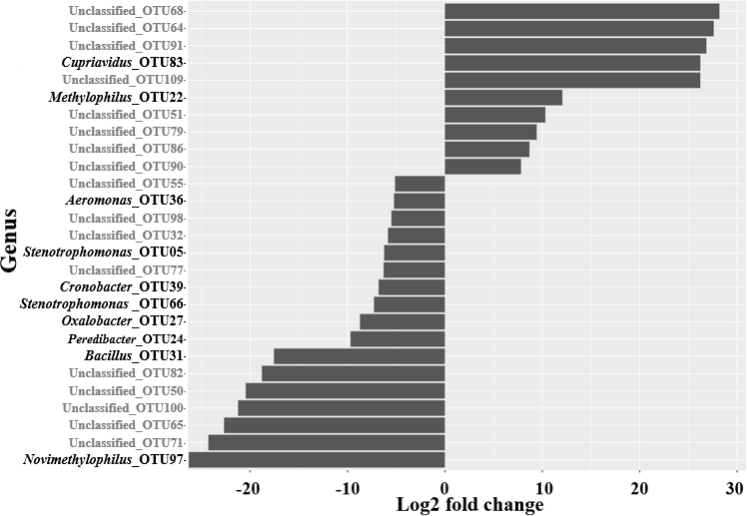
Difference in the abundance of some bacteria taxa in tsetse flies collected before and after 18 months of Tiny Targets implementation.

Differential abundance testing showed numerous OTUs that contributed to differences in the diversity between tsetse fly samples collected before and those collected over the vector control, with high log2-fold change >5 (Fig. 7). Twenty-seven OTUs, among which 12 classified at the genus level, were differentially abundant after 18 months. Some genera were completely absent from one of the sampling periods, like *Novimethylophilus* OTU097 from 0% to 0.01%, *Simkania* OTU071 from 0% to 0.08%, *Cupriavidus* OTU83 from 0.22% to 0%, and *Methylophilus* OTU022 from 1.97% to 0% (all *P*-values < 0.001).

## DISCUSSION

Blood-feeding arthropods harbor a wide variety of microbial taxa, although many questions remain about what factors shape the microbiome or to what extent they can be associated with the host biological features. The objective of this study was to determine a signature of modification of tsetse-associated microbiota during vector control using insecticide-impregnated Tiny Targets.

A total of 111 bacterial OTUs were detected, belonging to five phyla and 48 genera, providing a comprehensive update to the composition of *Glossina palpalis palpalis* microbiota in Campo, South Cameroon. Bacteria belonging to the phylum *Proteobacteria* were predominant in the present study with a mean relative abundance of 96.69%, which is similar to what was obtained in previous studies in Campo (22, 23, 50, 51). As previously observed, this was mainly due to the high relative abundance of the primary tsetse symbiont *Wigglesworthia,* which represented 71.76% of the total microbiome. This is not surprising since, as the obligate mutualist symbiont of tsetse flies, *Wigglesworthia* is essential to the survival of the fly by ensuring an important part of its immune response (52). *Wigglesworthia* is also vital for the maintenance of the fly’s population as the depletion of this bacteria by a specific antibiotic treatment results in sterile offspring (21, 53–55). Also, *Proteobacteria* represents the vast majority of bacteria found in association with insects; these taxa allow insects to manage their metabolism (56).

The main variation observed in the tsetse bacteria composition between sampling periods during the vector control was the overall increase in bacterial alpha diversity between 6 and 12 months of control, along with a decrease in the relative abundance of *Wigglesworthia* from 82.21% to 62.06%. The number of OTUs identified generally increased from 88 in flies before the vector control to 96 and 98 in flies collected after 12 and 18 months of vector control, respectively. Moreover, the relative abundance of many bacteria taxa increased significantly after 12 months of vector control, especially *Pelomonas* (0.27% to 8.36%), *Klebsiella* (1.23% to 3.18%), and *Curvibacter* (0.56% to 4.67%). These results can reflect the change in the tsetse fly microbiome population or the change in the tsetse population itself over the vector control, that is, most of the flies caught during the vector control are likely to be immigrants from neighboring areas not affected by the vector control as suggested by Melachio Tanekou et al. (33). An increase in bacterial taxa richness was recently reported by Juma et al. (57) after exposure of *Aedes albopictus* and *Culex pipiens* to malathion and permethrin, showing that adaptive microbes may facilitate the ability of hosts to match local environmental stressors as suggested by Henry et al. (58) or evolve novel functions faster than their hosts, providing adaptive abilities in a changing local environment (59). In the studied tsetse populations, we did not establish any clear link between these increased microbiome diversities and particular bacteria taxa known to be involved in insects’ ability to escape insecticide pressure. A more probable explanation of microbiome richness increase is that tsetse flies captured during the vector control are the ones reinvading the surface area covered by vector control from surrounding areas; these areas include the Campo national game reserve that borders the surface under vector control or the neighboring Equatorial Guinea, and these flies may harbor a different microbiome composition. This suggestion is reinforced by the good clustering of the flies from each capture period as shown in Fig. 4B and by the fact that most of the genera differentially abundant are unique to particular sample sets. Moreover, the greater bacterial diversity observed in males compared with females over the vector control period is linked to the fact that males have a greater dispersion, as already shown in tsetse population genetics data in the same population (60) or in other tsetse populations (61). However, *Curvibacter* and *Acinetobacter* showed a regular significant increase in relative abundance over the 18 months of vector control. Such an increase in abundance was shown in *Serratia marcescens* and *Pseudomonas protegens* harbored by wasp populations exposed to xenobiotics over generations, which was associated with metabolization of the pesticides (62). For now, there are no data available, to our knowledge, that support the potential implication of *Curvibacter* and/or *Acinetobacter* in maintaining the tsetse population during vector control.

### Conclusion

This study showed an increase in tsetse microbiome diversity in response to the Tiny Targets’ implementation. This increased diversity was due to new bacteria taxa identified in flies captured during the vector control but absent before and interestingly to other ones like *Curvibacter* and *Acinetobacter* whose abundance increased regularly over the vector control. These initial findings lay the groundwork for future investigations on the potential role that these specific microbes could play in tsetse population fitness or resilience capabilities against environmental or artificial selection factors like the insecticide-based Tiny Targets.

## Data Availability

All data generated or analysed during this study are included within the article and its additional files. The sequences generated have been deposited in the Sequence Read Archive (SAR) on GenBank database (study accession number: PRJNA837547).

## SUPPLEMENTAL MATERIAL

Fig. S1 spectrum.00935-24-s0001.tif Rarefaction curves showing the number of bacterial taxa identified and sequencing effort for each individual tsetse fly.

Table S1 spectrum.00935-24-s0002.docx Bacterial genera abundance in tsetse flies sampled before and during the vector control with insecticide impregnated Tiny Targets.

Table S2 spectrum.00935-24-s0003.xlsx Bacteria taxa abundances in different individual tsetse samples.

ASM does not own the copyrights to Supplemental Material that may be linked to, or accessed through, an article. The authors have granted ASM a non-exclusive, world-wide license to publish the Supplemental Material files. Please contact the corresponding author directly for reuse.
