# Supplementary figures and images for: Patterns of microbiome composition in tsetse fly Glossina palpalis palpalis during vector control using Tiny Targets in Campo, South Cameroon
Source: Microbiol Spectr. 2024 Sep 19;12(11):e00935-24. doi: 10.1128/spectrum.00935-24 (PMC11540164; doi:10.1128/spectrum.00935-24)

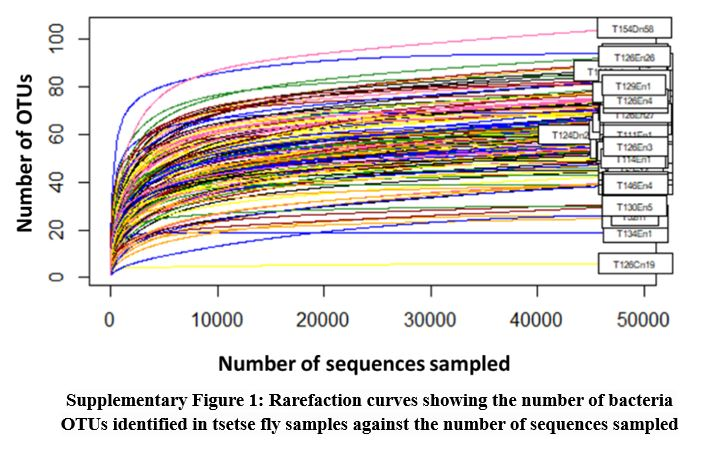

Supplement: Fig. S1 — Rarefaction curves showing the number of bacterial taxa identified and sequencing effort for each individual tsetse fly. [file spectrum.00935-24-s0001.tif]
