# Supplementary material for: Patterns of microbiome composition in tsetse fly Glossina palpalis palpalis during vector control using Tiny Targets in Campo, South Cameroon
Source: Microbiol Spectr. 2024 Sep 19;12(11):e00935-24. doi: 10.1128/spectrum.00935-24 (PMC11540164; doi:10.1128/spectrum.00935-24)
Supplement: Table S1 — Bacterial genera abundance in tsetse flies sampled before and during the vector control with insecticide impregnated Tiny Targets. [file spectrum.00935-24-s0002.docx]

**Supplementary Table S1:** Bacterial genera abundance according to sampling periods

| Genus | Before | 6months | 12months | 18months | p-value |
| --- | --- | --- | --- | --- | --- |
| *Wigglesworthia* | 82.041 | 82.216 | 62.061 | 66.450 | 0.019 |
| *Unclassified* | 4.589 | 4.790 | 10.141 | 3.134 | / |
| *Curvibacter* | 0.559 | 0.643 | 4.659 | 8.516 | 0.07 |
| *Pelomonas* | 0.268 | 4.644 | 8.361 | 0.613 | 0.077 |
| *Stenotrophomonas* | 0.352 | 0.079 | 0.099 | 9.457 | 0.154 |
| *Acinetobacter* | 1.247 | 1.588 | 1.351 | 3.798 | 0.037 |
| *Klebsiella* | 1.225 | 0.564 | 3.179 | 2.752 | 0.0396 |
| *Bacillus* | 2.657 | 3.423 | 0.206 | 0.639 | 0.058 |
| *Escherichia_Shigella* | 0.139 | 0.212 | 3.369 | 0.258 | 0.126 |
| *Pseudomonas* | 0.380 | 0.660 | 1.994 | 0.434 | 0.056 |
| *Staphylococcus* | 0.553 | 0.421 | 0.543 | 0.181 | 0.028 |
| *Undibacterium* | 0.079 | 0.046 | 0.463 | 0.603 | 0.061 |
| *Providencia* | 0.013 | 0.001 | 0.141 | 0.752 | 0.1228 |
| *Rugosibacter* | 0.009 | 0.166 | 0.641 | 0.058 | 0.098 |
| *Peredibacter* | 0.000 | 0.000 | 0.774 | 0.092 | 0.138 |
| *Methylophilus* | 1.929 | 0.001 | 0.028 | 0.231 | 0.1346 |
| *Burkholderia* | 0.102 | 0.004 | 0.015 | 0.404 | 0.108 |
| *Oxalobacter* | 0.001 | 0.000 | 0.000 | 0.419 | 0.1638 |
| *Enterococcus* | 0.085 | 0.014 | 0.144 | 0.228 | 0.0487 |
| *Aeromonas* | 0.079 | 0.057 | 0.146 | 0.149 | 0.0296 |
| *Orbus* | 0.293 | 0.000 | 0.273 | 0.000 | 0.079 |
| *Aquabacterium* | 0.058 | 0.022 | 0.231 | 0.056 | 0.069 |
| *Delfia_Variovorax* | 0.048 | 0.055 | 0.106 | 0.037 | 0.031 |
| *Massilia* | 0.285 | 0.019 | 0.013 | 0.065 | 0.1 |
| *Acidovorax* | 0.485 | 0.014 | 0.013 | 0.003 | 0.147 |
| *Cronobacter_Shigella* | 0.001 | 0.003 | 0.080 | 0.088 | 0.0735 |
| *Legionella* | 0.049 | 0.032 | 0.106 | 0.009 | 0.0534 |
| *Lactococcus* | 0.001 | 0.001 | 0.125 | 0.018 | 0.131 |
| *Streptococcus* | 0.235 | 0.012 | 0.042 | 0.013 | 0.105 |
| *Enhydrobacter* | 0.071 | 0.052 | 0.039 | 0.022 | 0.031 |
| *Stenotrophomonas_Luteimonas* | 0.000 | 0.031 | 0.051 | 0.035 | 0.046 |
| *Diaphorobacter_Comamonas* | 0.066 | 0.022 | 0.043 | 0.032 | 0.031 |
| *Exiguobacterium* | 0.215 | 0.023 | 0.019 | 0.009 | 0.111 |
| *Veillonella* | 0.363 | 0.000 | 0.001 | 0.003 | 0.1591 |
| *Simkania* | 0.000 | 0.029 | 0.069 | 0.005 | 0.099 |
| *Apilactobacillus* | 0.011 | 0.009 | 0.000 | 0.077 | 0.131 |
| *Novimethylophilus* | 0.000 | 0.000 | 0.073 | 0.009 | 0.164 |
| *Holophaga* | 0.087 | 0.001 | 0.053 | 0.002 | 0.09384 |
| *Lysinibacillus* | 0.271 | 0.003 | 0.000 | 0.000 | 0.192 |
| *Bacteria_unclassified* | 0.226 | 0.002 | 0.010 | 0.001 | 0.192 |
| *Aliicoccus* | 0.018 | 0.013 | 0.051 | 0.008 | 0.051 |
| *Thermaerobacter* | 0.001 | 0.000 | 0.000 | 0.074 | 0.191 |
| *Gp7_unclassified* | 0.000 | 0.029 | 0.034 | 0.000 | 0.092 |
| *Cupriavidus* | 0.213 | 0.000 | 0.000 | 0.000 | 0.195 |
| *Weissella* | 0.003 | 0.009 | 0.029 | 0.017 | 0.04 |
| *Lysobacter* | 0.000 | 0.000 | 0.006 | 0.045 | 0.1622 |
| *Aerococcus* | 0.056 | 0.005 | 0.017 | 0.008 | 0.082 |
| *Methyloversatilis* | 0.128 | 0.000 | 0.001 | 0.006 | 0.1803 |
| *Chromohalobacter* | 0.000 | 0.003 | 0.039 | 0.000 | 0.1748 |
| *Lactobacillus* | 0.000 | 0.040 | 0.001 | 0.001 | 0.181 |
| *Alishewanella* | 0.000 | 0.000 | 0.002 | 0.040 | 0.1812 |
| *Psychrobacter* | 0.000 | 0.000 | 0.004 | 0.032 | 0.1634 |
| *Sphingosinithalassobacter* | 0.000 | 0.000 | 0.006 | 0.029 | 0.145 |
| *Rheinheimera* | 0.105 | 0.000 | 0.002 | 0.000 | 0.19 |
| *Hyphomicrobium* | 0.001 | 0.012 | 0.017 | 0.001 | 0.073 |
| *Jeotgalicoccus* | 0.003 | 0.004 | 0.015 | 0.009 | 0.034 |
| *Gemella* | 0.091 | 0.001 | 0.002 | 0.000 | 0.1865 |
| *Chryseomicrobium* | 0.028 | 0.003 | 0.006 | 0.013 | 0.055 |
| *Kurthia* | 0.002 | 0.000 | 0.000 | 0.027 | 0.1747 |
| *Arsenophonus* | 0.031 | 0.001 | 0.016 | 0.000 | 0.099 |
| *Perlucidibaca* | 0.000 | 0.000 | 0.025 | 0.000 | 0.195 |
| *Geothrix* | 0.041 | 0.000 | 0.013 | 0.000 | 0.12911 |
| *Alloiococcus* | 0.000 | 0.005 | 0.016 | 0.001 | 0.116 |
| *Coxiella* | 0.073 | 0.000 | 0.000 | 0.000 | 0.195 |
| *Neisseria* | 0.057 | 0.000 | 0.000 | 0.001 | 0.1911 |
| *Alkanindiges* | 0.000 | 0.000 | 0.000 | 0.017 | 0.1955 |
| *Floricoccus* | 0.002 | 0.000 | 0.007 | 0.006 | 0.0557 |
| *Silvanigrella* | 0.001 | 0.004 | 0.009 | 0.000 | 0.089 |
| *Haemophilus* | 0.030 | 0.000 | 0.000 | 0.000 | 0.195 |
| *Leuconostoc* | 0.000 | 0.008 | 0.000 | 0.000 | 0.195 |
| *Lactiplantibacillus* | 0.004 | 0.001 | 0.006 | 0.000 | 0.069 |
| *Frischella* | 0.000 | 0.000 | 0.001 | 0.007 | 0.16212 |
| *Facklamia* | 0.002 | 0.000 | 0.006 | 0.000 | 0.124 |
| *Hydrogenophaga* | 0.021 | 0.000 | 0.000 | 0.000 | 0.195 |
| *Pseudescherichia* | 0.000 | 0.000 | 0.005 | 0.000 | 0.1629 |
| *Aquitalea* | 0.015 | 0.000 | 0.000 | 0.000 | 0.16309 |
| *Macrococcus* | 0.000 | 0.000 | 0.000 | 0.004 | 0.163 |
